# Whole Transcriptome Profiling of Adrenocortical Tumors Using Formalin-Fixed Paraffin-Embedded Samples

**DOI:** 10.3389/fendo.2022.808331

**Published:** 2022-02-03

**Authors:** Norifusa Iwahashi, Hironobu Umakoshi, Masatoshi Ogata, Tazuru Fukumoto, Hiroki Kaneko, Eriko Terada, Shunsuke Katsuhara, Naohiro Uchida, Katsuhiko Sasaki, Maki Yokomoto-Umakoshi, Yayoi Matsuda, Ryuichi Sakamoto, Yoshihiro Ogawa

**Affiliations:** ^1^ Department of Medicine and Bioregulatory Science, Graduate School of Medical Sciences, Kyushu University, Fukuoka, Japan; ^2^ Clinical Laboratory Department, Kyushu Pro Search Limited Liability Partnership, Fukuoka, Japan

**Keywords:** whole transcriptome profiling, RNA sequencing (RNAseq), formalin-fixed paraffin-embedded samples (FFPE samples), adrenal diseases, adrenocortical tumors

## Abstract

Whole transcriptome profiling is a promising technique in adrenal studies; however, whole transcriptome profiling of adrenal disease using formalin-fixed paraffin-embedded (FFPE) samples has to be further explored. The aim of this study was to evaluate the utility of transcriptome data from FFPE samples of adrenocortical tumors. We performed whole transcriptome profiling of FFPE and fresh frozen samples of adrenocortical carcinoma (ACC, n = 3), aldosterone-producing adenoma (APA, n = 3), and cortisol-producing adenoma (CPA, n = 3), and examined the similarity between the transcriptome data. We further examined whether the transcriptome data of FFPE samples could be used to distinguish tumor types and detect marker genes. The number of read counts was smaller in FFPE samples than in fresh frozen samples (P < 0.01), while the number of genes detected was similar (P = 0.39). The gene expression profiles of FFPE and fresh frozen samples were highly correlated (r = 0.93, P < 0.01). Tumor types could be distinguished by consensus clustering and principal component analysis using transcriptome data from FFPE samples. In the differential expression analysis between ACC and APA-CPA, known marker genes of ACC (e.g., *CCNB2*, *TOP2A*, and *MAD2L1*) were detected in FFPE samples of ACC. In the differential expression analysis between APA and CPA, known marker genes of APA (e.g., *CYP11B2*, *VSNL1*, and *KCNJ5*) were detected in the APA of FFPE samples. The results suggest that FFPE samples may be a reliable alternative to fresh frozen samples for whole transcriptome profiling of adrenocortical tumors.

## Introduction

The adrenal cortex produces a variety of steroid hormones and maintains metabolic homeostasis (1). Aldosterone, produced in the zona glomerulosa, is involved in the regulation of both the Na/K balance and blood pressure, while cortisol, produced in the zona fasciculata, plays an important role in the regulation of glucose metabolism and the immune response. Some adrenocortical adenomas produce these steroid hormones inappropriately and cause a variety of clinical syndromes. Aldosterone-producing adenoma (APA) causes primary aldosteronism, and cortisol-producing adenoma (CPA) causes Cushing’s syndrome (2, 3). Adrenocortical carcinoma (ACC) is a rare malignant tumor that arises in the adrenal cortex and has a very poor prognosis (4). In the clinical management of adrenocortical tumors, it is important to distinguish between these tumors, especially ACC and adenoma, although they can have overlapping clinical and pathologic findings that can be difficult to distinguish. Previous transcriptome studies have shown that APA, CPA, and ACC have distinct gene expression profiles, suggesting that transcriptome profiling can contribute to the accurate diagnosis of adrenocortical tumors (5, 6). Furthermore, transcriptome profiling is also a promising technique for elucidating the pathogenesis of adrenal diseases in terms of endocrine function and adrenal differentiation (7, 8).

Molecular profiling, including transcriptomics, usually requires fresh frozen tissue samples. However, these are difficult to obtain for most adrenocortical tumors because fresh frozen tissue is rarely preserved in routine clinical practice. This is a major obstacle to transcriptome studies of adrenocortical tumors. However, formalin-fixed paraffin-embedded (FFPE) samples are relatively easy to obtain, but the nucleic acids are degraded and less high-quality DNA and RNA can be extracted compared with fresh frozen samples (9, 10). In recent years, advances in sequencing technology have made it possible to perform molecular profiling using nucleic acids extracted from FFPE samples, and techniques for the genomic profiling of tumors using DNA extracted from FFPE samples have been established and are being used clinically (11). Whole transcriptome profiling using FFPE samples has been reported to be useful in malignant tumors such as hepatocellular carcinoma and breast cancer (12, 13). However, it remains unknown whether whole transcriptome profiling of adrenal disease using FFPE samples can be successfully performed.

In this study, we performed whole transcriptome profiling using FFPE samples of adrenocortical tumors (ACC, APA, and CPA) and compared the results with those of fresh frozen samples. We demonstrated the utility of FFPE samples in the whole transcriptome profiling of adrenocortical tumors.

## Materials and Methods

### Sample Preparation

We used ACC (n = 3), APA (n = 3), and CPA (n = 3) tumor tissues obtained from adrenocortical tumor patients operated at Kyushu University Hospital from 2017 to 2020. Diagnosis was made based on clinical symptoms, imaging features, and postoperative histopathological data. Each tumor was stored by both FFPE and fresh frozen after surgery. A portion of each tumor was collected in microtubes immediately after surgical removal and stored in a deep freezer (-80°C) (fresh frozen sample). The rest of the tumor was formalin-fixed and paraffin-embedded (FFPE sample). FFPE samples were stored at room temperature and fresh frozen samples were stored at -80°C for 1-4 years until RNA extraction. The detailed characteristics of each tumor sample were summarized in **Supplementary Table 1**.

### RNA Isolation

FFPE samples were sectioned into 10 μm slices and only the tumor area was collected under visual observation. Total RNA was extracted from the collected tumor using the Maxwell RSC RNA FFPE Kit (Promega) according to the manufacturer’s instructions. Total RNA from fresh frozen samples was extracted using TRIzol reagent (Thermo Fisher) according to the manufacturer’s instructions. The RNA yield and quality were determined by UV absorption using a NanoDrop 2000 spectrophotometer. The RNA fragment size of FFPE samples was analyzed using the RNA 6000 Pico Kit (Agilent Technologies) running on the 2100 Bioanalyzer. Similarly, the RNA fragment size of fresh frozen samples was analyzed using the RNA 6000 Nano Kit. RNA integrity number (RIN) values and DV200 (the percentage of RNA fragments above 200 nucleotides in length) were obtained by running Bioanalyzer according to the manufacturer’s instructions. RNA quality was assessed using RIN and DV200 values.

### Library Preparation, Sequencing, and Alignment

Ribosomal RNA was removed from 100 ng of total RNA of each sample using the NEBNext rRNA Depletion Kit (Human/Mouse/Rat) (NEB, E6310). Libraries were then prepared using the NEBNext Ultra II Directional RNA Library Prep Kit for Illumina (NEB, E7760). Libraries were quantified using the Bioanalyzer DNA-sensitivity kit (Agilent, 5067-4626) and sequenced on the NextSeq 500 (Illumina) using a 36 cycle, paired-end protocol providing approximately 33 million reads per sample. Base call files were converted to the FASTQ format using Bcl2Fastq (Illumina). Using the CLC genomics workbench (v10.1.1), all reads were aligned to the reference genome (Human, hg19), and gene expression was quantified.

### Data Analysis

We used R (version 4.1.1) to perform the data analysis. Gene expression data were used as inputs for the R package edgeR (14). Genes with low expression were excluded using the “filterByExpr” function of edgeR. The gene counts were normalized by applying the trimmed mean of M-values (TMM) normalization method using the “calcNormFactors” and “cpm” functions of edgeR. The resulting log2 counts per million (logCPM) were used as inputs for downstream analyses (e.g., sample-to-sample correlation and consensus clustering). Consensus clustering (with the number of clusters “k” evaluated for k = 2–6) was performed using the R package ConsensusClusterPlus (15). Fifty iterations were performed with a sample inclusion probability of 0.8 and an item inclusion probability of 1. The number of clusters was selected based on the inspection of the delta area plot. Principal component analysis was performed using the “prcomp” function of R package stats (16–18). Differentially expressed genes (DEGs) between adrenocortical tumors (ACC and the other types, or APA and CPA) were detected using the “lmfit” and “eBayes” functions of the R package limma (19, 20). The Benjamini-Hochberg method was used to correct for multiple comparisons. DEGs were defined as genes with an absolute value of log2 fold-change (logFC) greater than 2 and an adjusted P value (adj. P) of less than 0.05. KEGG pathway analysis was performed using the R package pathfindR (21). The “run_pathfindR” function was used with default parameters to identify the enriched KEGG pathways. The “score_terms” function was then used to calculate the agglomerated z score of each enriched pathway per sample.

## Results

### Differences in Transcriptome Data Obtained From FFPE and Fresh Frozen Samples

We extracted RNA from a total of nine FFPE and nine fresh frozen samples from three ACC, three APA, and three CPA cases. Total RNA extracted from FFPE samples had lower RNA yield and quality parameters compared to those from fresh frozen samples (RNA concentration [mean 36.0 vs 464.5 μL, Wilcoxon rank sum test: P value < 0.01], RIN [mean 2.2 vs 7.9, P value < 0.01], DV200 [mean 47.6 vs 71.0, P value < 0.01]). To examine the effect of storage period on RNA yield and quality for each storage method, we divided the samples into two groups by the median storage period from surgery to RNA extraction and compared the two groups. There was no significant difference in the RNA yield and quality (**Supplementary Figure 1**).

**Figure 1:**
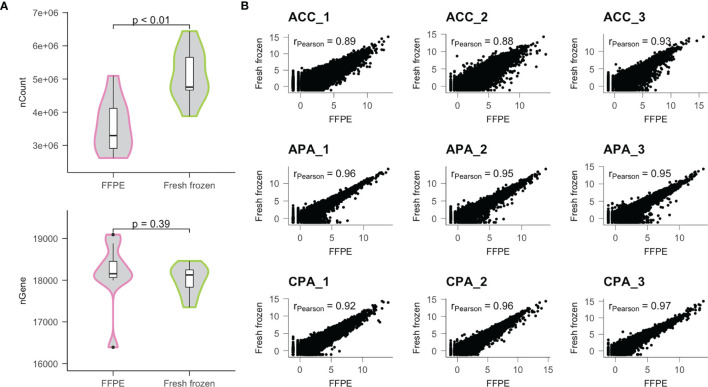
Comparison of transcriptome data obtained from FFPE and fresh frozen samples. **(A)** Distributions of the number of read counts (nCount) and the number of genes detected (nGene) were compared between FFPE and fresh frozen samples. **(B)** Results of correlation analysis between FFPE and fresh frozen samples for each patient. r; Pearson’s correlation coefficient.

Next, we performed RNA sequencing (RNA-seq) using the extracted RNA. The number of raw reads obtained from FFPE samples was similar to those from frozen samples (mean 3.30E^7^ vs 3.29E^7^, P value = 0.86), but the percentage of mapped reads of FFPE samples was less than those of fresh frozen samples (mean 82.9% vs 87.7%, P value = 0.02) (**Supplementary Table 1**). Consequently, the read counts obtained from FFPE samples were less than those from the fresh frozen samples (mean 3.58E^6^ vs 5.13E^6^, P value < 0.01) (**Figure 1A**). In contrast, the number of unique genes detected in FFPE samples was similar to those in fresh frozen samples (mean 18160 vs 18001, P value = 0.39).

Using normalized gene expression data, we examined the correlation between samples. In each case, the FFPE and fresh frozen samples were highly correlated (mean Pearson’s correlation coefficient [r] = 0.93) (**Figure 1B**). Therefore, we found that similar gene expression profiles could be obtained using FFPE samples, although the amount of detectable transcriptome was lower than that of fresh frozen samples.

### Distinction Between ACC, APA, and CPA Using Transcriptome Data From FFPE Samples

**Figure 2:**
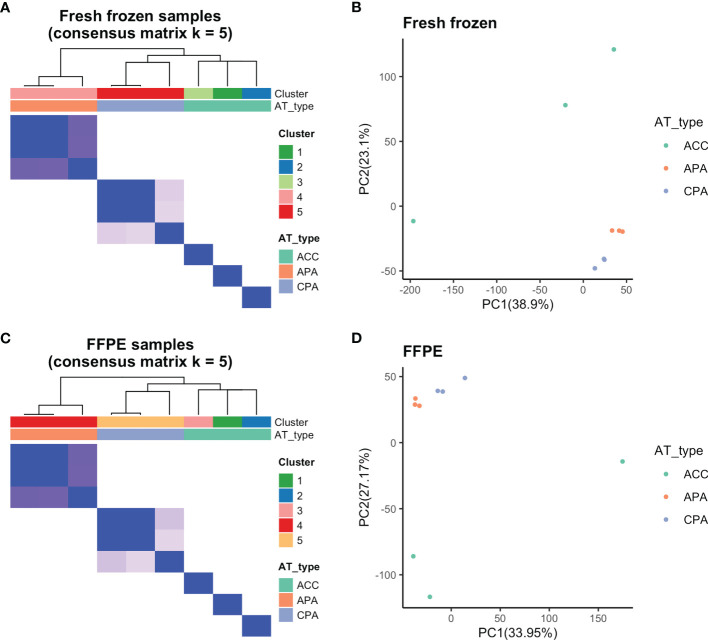
Results of consensus clustering and principal component analysis. **(A)** Fresh frozen samples were divided into five clusters by consensus clustering (Cluster 1-3: ACC, Cluster 4: APA, Cluster 5: CPA samples). **(B)** Principal component analysis of fresh frozen samples confirmed separation based on tumor types. **(C)** By consensus clustering, FFPE samples were also divided into five clusters (Cluster 1-3: ACC, Cluster 4: APA, Cluster 5: CPA samples) similar to fresh frozen samples. **(D)** Principal component analysis of FFPE samples confirmed separation based on tumor types.

We examined whether transcriptome data from FFPE samples could be used to distinguish between ACC, APA, and CPA. First, we validated whether the adrenocortical tumor types (ACC, APA, and CPA) could be separated at the transcriptome level using fresh frozen samples. Using consensus clustering, fresh frozen samples were classified into five clusters. APA and CPA were each classified into one cluster, and ACC was classified into one cluster per case (**Figure 2A** **)**. Principal component analysis of fresh frozen samples was performed and visualized on two dimensions, with principal components 1 and 2 as the axes. APA and CPA each formed one cluster, whereas ACC was scattered in each case (**Figure 2B**). These results confirmed that the three types of tumors used in this study (ACC, APA, and CPA) had distinct gene expression profiles. In addition, ACC was considered to have a large difference in gene expression profiles among the cases.

Further, we performed consensus clustering and principal component analysis of the transcriptome data of FFPE samples. The FFPE samples were classified into five clusters by consensus clustering. APA and CPA were each classified into one cluster, and ACC was classified into one cluster per case (**Figure 2C**). Principal component analysis of FFPE samples was performed and visualized on two dimensions with principal components 1 and 2 as the axes. APA and CPA each formed one cluster, whereas ACC was scattered in each case (**Figure 2D**). The results of consensus clustering and principal component analysis of FFPE samples were both similar to those of fresh frozen samples. Therefore, we found that it was possible to distinguish between ACC, APA, and CPA using transcriptome data from FFPE samples.

### Detection of Adrenocortical Tumor Marker Genes Using Transcriptome Data From FFPE Samples

**Figure 3:**
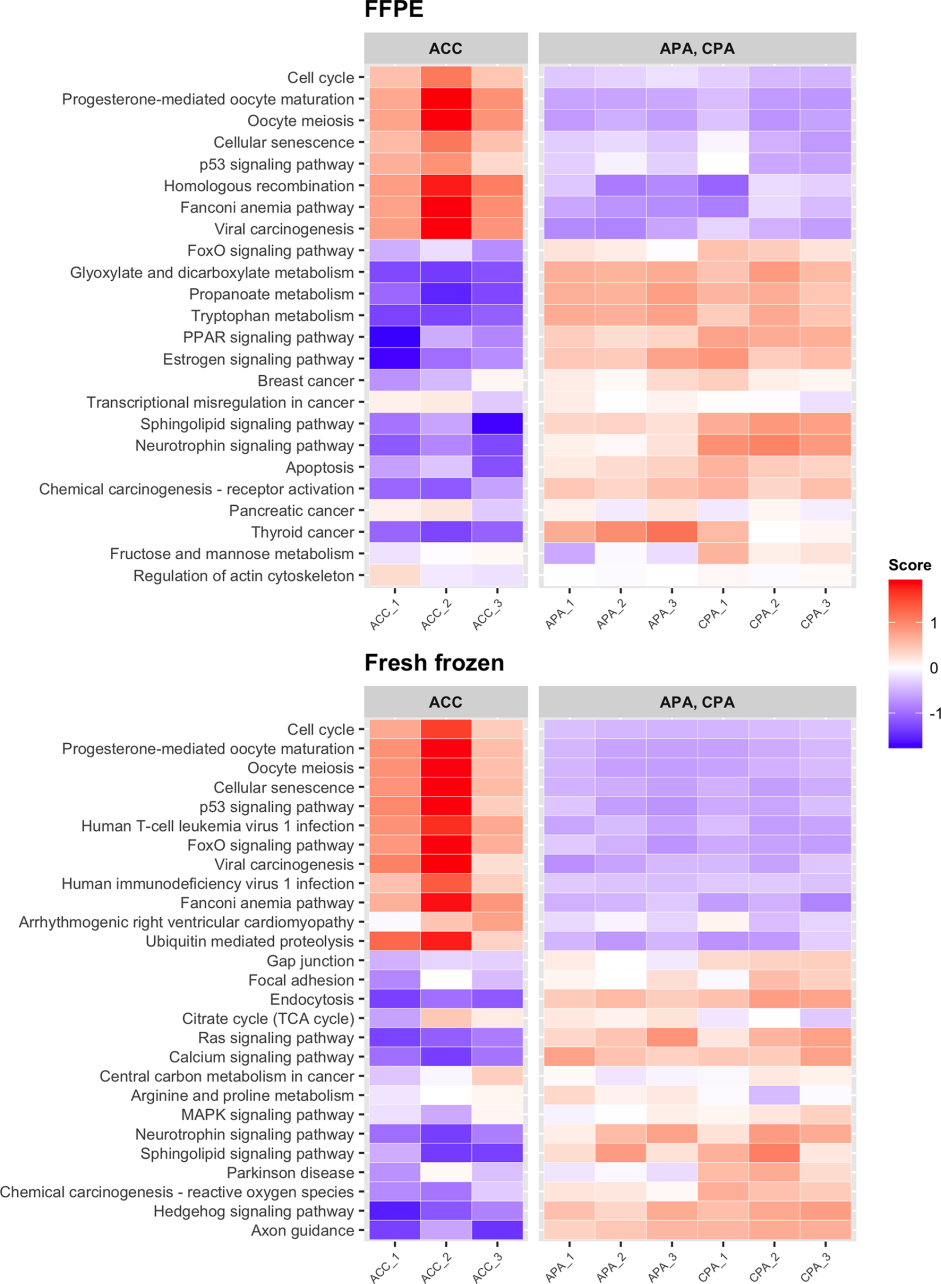
Heatmap showing the results of KEGG pathway analysis of DEGs detected between ACC and APA-CPA by each storage type. Score; the agglomerated z score of each enriched KEGG pathway per sample.

We examined whether transcriptome data from FFPE samples could be used to detect previously reported adrenocortical tumor marker genes. First, we performed differential expression analysis between ACC and APA-CPA for each storage method (FFPE and fresh frozen). In FFPE samples of ACC, 402 DEGs were detected (161 upregulated and 241 downregulated). In fresh frozen samples of ACC, 356 DEGs were detected (148 upregulated and 208 downregulated). 193 DEGs were common between FFPE and fresh frozen samples (76 upregulated and 117 downregulated). The upregulated DEGs in FFPE samples of ACC included genes previously reported to be upregulated in ACC, such as *CCNB2* (encoding cyclin B2), *TOP2A* (encoding DNA topoisomerase II alpha), and *MAD2L1* (encoding mitotic arrest deficient 2 like 1) (**Table 1** and **Supplementary Table 2**) (22–24). These genes were also included in the upregulated DEGs in fresh frozen samples of ACC. *IGF2* (encoding insulin like growth factor 2), commonly used as a marker for ACC (25, 26), was not identified as an upregulated DEG in either FFPE or fresh frozen samples. This may be due to the low expression of *IGF2* in both FFPE and fresh frozen samples of case ACC_2 (**Supplementary Figure 2**). KEGG pathway analysis showed that upregulated DEGs in FFPE samples of ACC were enriched in tumor-related pathways including “cell cycle” and “p53 signaling pathway” (**Figure 3**). The upregulated DEGs in fresh frozen samples of ACC were also enriched in similar tumor-related pathways. Therefore, it was confirmed that the transcriptome data from FFPE samples could be used to detect the genes characteristic of ACC.

**Table 1:** DEGs between ACC and APA-CPA.

Gene	FFPE sample		Fresh frozen sample		Gene description
	logFC	adj.P	logFC	adj.P	
ANLN	4.28	1.28E-02	4.44	3.99E-02	anillin actin-binding protein
ASPM	4.28	5.65E-03	4.65	1.47E-02	assembly factor for spindle microtubules
FOXM1	4.26	6.93E-03	3.99	1.14E-02	forkhead box M1
RRM2	4.02	1.37E-02	4.44	4.29E-02	ribonucleotide reductase regulatory subunit M2
DTL	3.77	1.87E-02	3.91	2.77E-02	Denticle-less E3 ubiquitin-protein ligase homolog
CCNB2	3.72	1.26E-02	4.49	5.09E-03	cyclin B2
TOP2A	3.71	6.92E-03	4.57	2.31E-02	DNA topoisomerase II alpha
TPX2	3.67	2.66E-02	3.85	2.16E-02	TPX2 microtubule nucleation factor
KIAA0101	3.41	1.19E-02	4.01	9.28E-03	PCNA clamp-associated factor

Showing genes related to ACC. Upregulated genes in ACC in the study by Giordano et al. were used as reference.

The higher the logFC, the higher the expression in ACC than APA-CPA.

**Figure 4:**
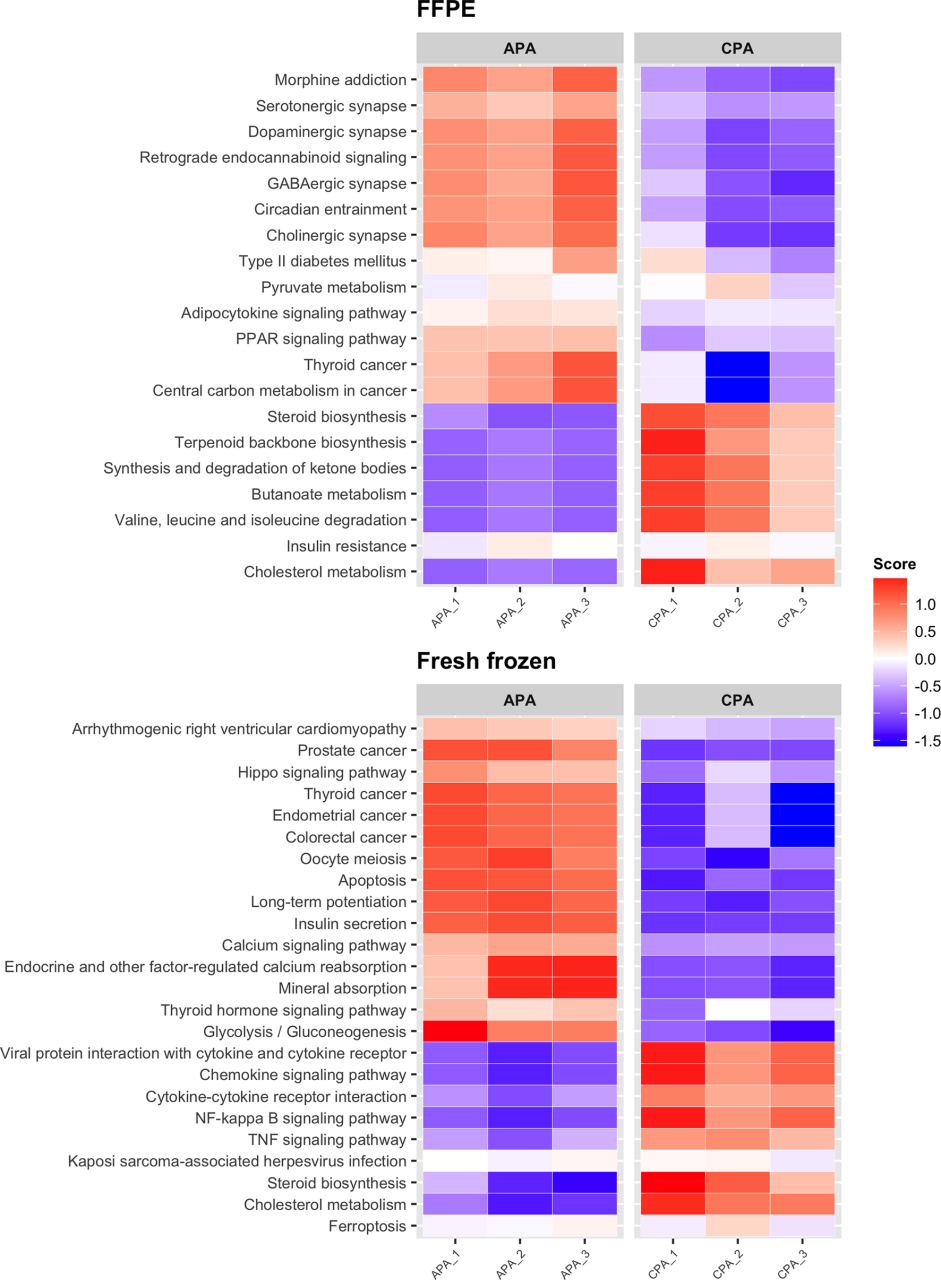
Heatmap showing the results of KEGG pathway analysis of DEGs between APA and CPA by each storage type. Score; the agglomerated z score of each enriched KEGG pathway per sample.

Next, we performed differential expression analysis between APA and CPA for each storage method (FFPE and fresh frozen). In FFPE samples of APA, 135 upregulated DEGs were detected. In fresh frozen samples of APA, 106 upregulated DEGs were detected. Forty-seven upregulated DEGs were common between FFPE and fresh frozen samples. The upregulated DEGs in FFPE samples of APA included genes reported to be associated with APA, such as *CYP11B2* (encoding cytochrome P450 family 11 subfamily B member 2, also known as aldosterone synthase), *KCNJ5* (encoding potassium inwardly rectifying channel subfamily J member 5), *VSNL1* (encoding visinin like 1), *CALN1* (encoding calneuron 1), and *HTR4* (encoding 5-hydroxytryptamine receptor 4) (**Table 2** and **Supplementary Table 3**) (27–31). These genes were also included in the upregulated DEGs in fresh frozen samples of APA. While in FFPE samples of CPA, 115 upregulated DEGs were detected. In fresh frozen samples of CPA, 97 upregulated DEGs were detected. Forty-eight upregulated DEGs were common between FFPE and fresh frozen samples. The upregulated DEGs in FFPE samples of CPA included genes reported to be upregulated in CPA, such as *FATE1* (fetal and adult testis expressed 1), *PITX1* (encoding paired like homeodomain 1) and *CXCL2* (encoding C-X-C motif chemokine ligand 2) (7, 32). KEGG pathway analysis showed that upregulated DEGs in FFPE samples of APA were enriched in pathways such as “serotonergic synapse” and “circadian entrainment”, while DEGs in fresh frozen samples of APA were enriched in pathways such as “calcium signaling pathway” and “hippo signaling pathway” (**Figure 4**). The common upregulated DEGs between FFPE samples and fresh frozen samples were enriched in the pathway of “aldosterone synthesis and secretion” (**Supplementary Figure 3**). The upregulated DEGs in FFPE samples of CPA were enriched in metabolism-related pathways such as “steroid biosynthesis” and “cholesterol metabolism” (**Figure 4**). The upregulated DEGs in fresh frozen samples of CPA were also enriched in similar metabolism-related pathways. Thus, it was confirmed that transcriptome data from FFPE samples could be used to detect the characteristic genes of APA and CPA.

**Table 2:** DEGs between APA and CPA.

Gene	FFPE sample		Fresh frozen sample		Genn	Reference
	logFC	adj.P	logFC	adj.P		
CYP11B2	5.00	1.14E-02	7.00	1.41E-02	cytochrome P450 family 11 subfamily B member 2	Bassett MH et al. J Clin Endocrinol Metab. (27);90 (9):5446-5455.
VSNL1	3.57	8.92E-03	4.31	1.80E-02	visinin like 1	Williams TA et al. Hypertension. (28);59 (4):833-839.
CALN1	3.56	8.19E-03	5.81	3.87E-03	calneuron 1	Kobuke K et al. Hypertension. (29);71 (1):125-133.
HTR4	3.23	2.74E-03	4.54	4.92E-03	5-hydroxytryptamine receptor 4	Ye P et al. J Endocrinol. (30);195 (1):39-48.
KCNJ5	2.98	4.55E-03	4.27	2.21E-02	potassium inwardly-rectifying channel subfamily J member 5	Choi M et al. Science. (31);331 (6018):768-772.

Showing genes related to APA.

The higher the logFC, the higher the expression in APA than CPA.

## Discussion

To evaluate the utility of transcriptome data obtained from FFPE samples in adrenocortical tumors, we compared transcriptome data from FFPE samples of ACC, APA, and CPA with those from fresh frozen samples of the same tumors. Transcriptome data from FFPE and fresh frozen samples showed a high degree of similarity. Using transcriptome data from FFPE samples, we were able to distinguish between ACC, APA, and CPA and detect the marker genes. Our study is the first to show that FFPE samples may be an alternative to fresh frozen samples for whole transcriptome profiling of adrenocortical tumors.

Recently, transcriptome profiling using FFPE samples has been performed in a variety of tissues (12, 13), but few studies have validated the accuracy of gene expression profiles obtained from FFPE samples using fresh frozen samples of the same tissue. Hedegaard et al. reported a comparative study of RNA-seq using FFPE and fresh frozen samples from the same tissue of six different human tissue types (bladder, prostate, and colon carcinoma; liver and colon normal tissue; reactive tonsil) (33). Shohdy et al. reported a comparative study of RNA-seq using FFPE and fresh frozen samples from the same tumors of seven different tumors (urothelial cancer, gastroesophageal junction adenocarcinoma, oligodendroglioma, cancer of unknown primary, leiomyosarcoma, papillary thyroid cancer, and colorectal cancer) in 11 patients (34). In this study, we showed that gene expression profiles from FFPE samples were highly similar to those from fresh frozen samples of the same adrenocortical tumors (ACC, APA, and CPA).

In transcriptome profiling using FFPE samples, targeted RNA-seq is often used because of the low yield and quality of RNA extracted from such samples (35, 36). Plaska et al. reported that targeted RNA-seq (194 target genes) using FFPE samples of adrenocortical tumors (ACC, APA, and CPA) could distinguish between benign and malignant tumors (36). However, targeted RNA-seq is not suitable for comprehensive genetic analysis (e.g., detection of novel pathogenic genes) because it restricts the genes that can be analyzed. In this study, we used whole transcriptome RNA-seq rather than targeted RNA-seq to obtain expression profiles of a large number of genes in each tumor (average number of genes detected in FFPE samples: 18001). We thus demonstrated that it is possible to distinguish ACC, APA, and CPA and detect their marker genes using transcriptome data from FFPE samples. Our results support the possible utility of whole transcriptome profiling using FFPE samples of adrenocortical tumors.

In the consensus clustering and principal component analysis of this study, APA and CPA each formed one cluster, and ACC differed greatly among cases. This result was similar for both FFPE and fresh frozen samples. The variation in ACC may be due to the different tumor traits in each case. Alternatively, it may be due to technical issues such as the storage conditions of each sample and the storage period until RNA extraction. A larger number of cases would be needed to examine the differences in tumor traits in ACC.

In the differential expression analysis of this study, it was possible to detect the marker genes of each adrenocortical tumor using FFPE samples. There were also differences in the DEGs that could be detected using FFPE samples and fresh frozen samples. In FFPE samples of ACC, genes such as *SPP1*, *PBK*, and *UBE2C* could not be detected. In FFPE samples of APA, genes such as *LGR5*, *HOPX*, and *ATP2B3* could not be detected. The read counts obtained from FFPE samples were lower than those of fresh frozen samples, which may result in the lower detection sensitivity of relatively low expression genes. When using FFPE samples for whole transcriptome profiling, a larger number of samples may be required compared to fresh frozen samples.

The use of FFPE samples for whole transcriptome profiling has advantages other than the ease of sample collection. FFPE samples are more suitable for morphological observation than fresh frozen samples, making it easier to collect transcriptomes from small regions of interest following microdissection. This may be applied, for example, to examine each layer of the adrenal cortex (which is composed of three layers) or small lesions such as aldosterone-producing cell clusters (presumed to be precursor lesions of APA) (37).

The limitation of this study is that the storage period of FFPE samples was relatively short (4 years at the longest). The longer the storage period, the lower the yield and quality of RNA, which may make it difficult to perform gene expression profiling equivalent to that using fresh frozen samples (38). Studies using FFPE samples with longer storage periods are required to validate our results. Another limitation is the lack of comparison between adrenocortical tumors and normal adrenocortical tissue adjacent to the tumors. In examining the intrinsic properties of each adrenocortical tumor, the normal adrenocortical tissue may be the ideal comparison target.

In conclusion, in this study, we demonstrated the utility of gene expression profiling of adrenocortical tumors using FFPE samples. FFPE samples are relatively easier to obtain, thus allowing large-scale adrenocortical tumor transcriptome studies.

## Data Availability Statement

The datasets presented in this study can be found in online repositories. The names of the repository/repositories and accession number(s) can be found below: https://www.ncbi.nlm.nih.gov/sra/PRJNA787399, PRJNA787399.

## Ethics Statement

The studies involving human participants were reviewed and approved by the medical ethics committee of the Kyushu University Hospital (approval #21025-00). The patients/participants provided their written informed consent to participate in this study.

## Author Contributions

NI wrote the initial draft. HU and YO edited the manuscript. NI analyzed the results. HU and YO managed this study. HU contributed to data interpretation. MO, TF, HK, ET, SK, NU, KS, MY-U, YM, and RS curated the data and provided critical feedback and helped shape the research, analysis, and manuscript. All authors contributed to the article and approved the submitted version.

## Funding

This work was supported by grant “KAKENHI 20K17493”, grant “The Uehara Memorial Foundation”, grant “Secom Science and Technology Foundation”, grant “Kaibara Morikazu Medical Science Promotion Foundation”, grant “Takeda Science Foundation”, grant “KAKENHI 20K16525”, grant “KAKENHI 20K17514”, grant “KAKENHI 20K21604”, and “The Mitsubishi Foundation”.

## Conflict of Interest

KS was employed by Kyushu Pro Search Limited Liability Partnership.

The remaining authors declare that the research was conducted in the absence of any commercial or financial relationships that could be construed as a potential conflict of interest.

## Publisher’s Note

All claims expressed in this article are solely those of the authors and do not necessarily represent those of their affiliated organizations, or those of the publisher, the editors and the reviewers. Any product that may be evaluated in this article, or claim that may be made by its manufacturer, is not guaranteed or endorsed by the publisher.

## Supplementary Material

Supplementary Figure 1 Comparison of RNA yield and quality parameters between two storage period groups. FFPE samples were divided into two groups by 340 days, the median storage period. Similarly, fresh-frozen samples were divided by 427 days.

Supplementary Figure 2 Distribution of the logCPM values of *IGF2*. Dots; each sample.

Supplementary Figure 3 Heatmap showing the results of KEGG pathway analysis of DEGs between APA and CPA (common to FFPE and fresh frozen samples). Score; the agglomerated z score of each enriched KEGG pathway per sample.
