# Supplementary figures and images for: Whole Transcriptome Profiling of Adrenocortical Tumors Using Formalin-Fixed Paraffin-Embedded Samples
Source: Front Endocrinol (Lausanne). 2022 Feb 3;13:808331. doi: 10.3389/fendo.2022.808331 (PMC8850780; doi:10.3389/fendo.2022.808331)

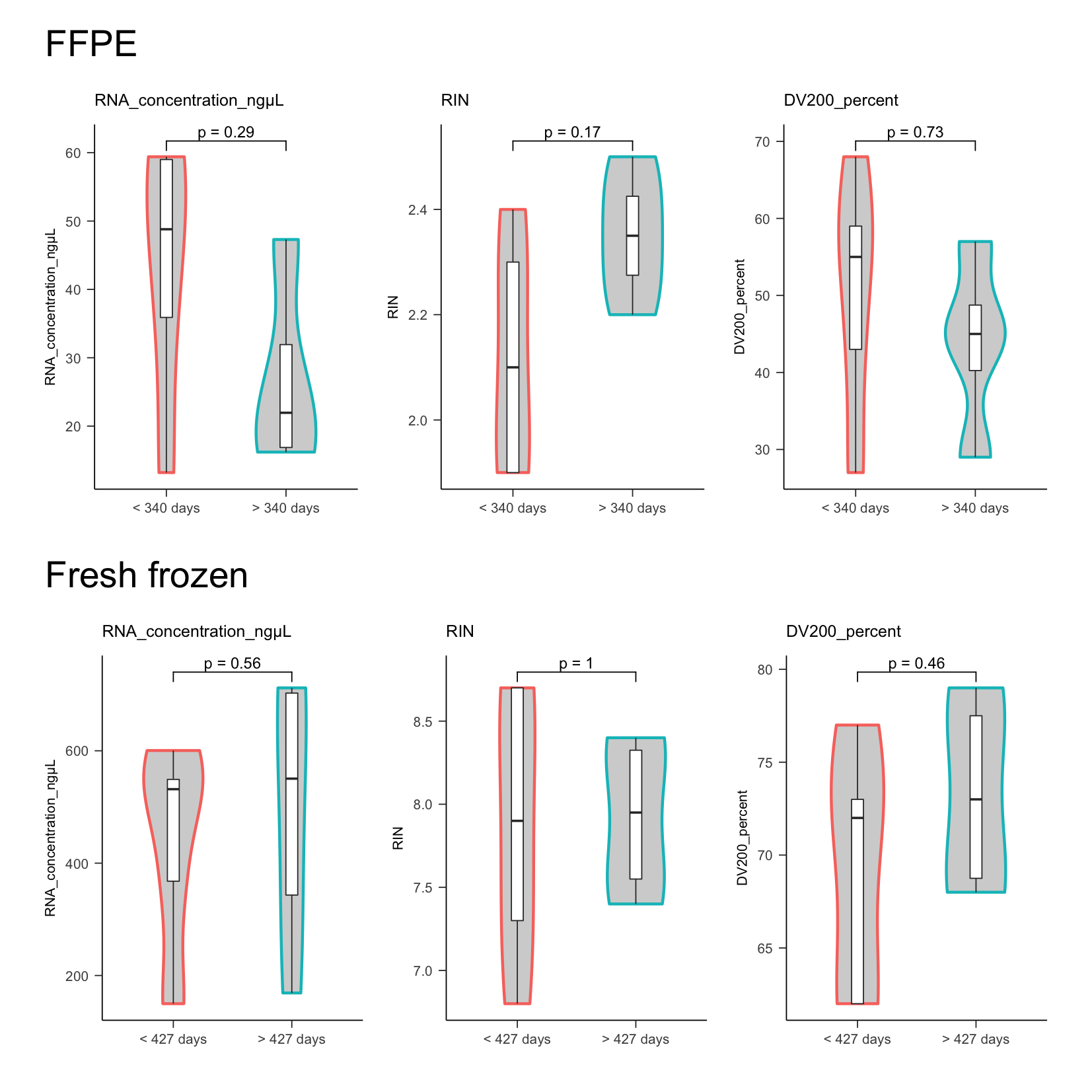

Supplement: Supplementary Figure 1 — Comparison of RNA yield and quality parameters between two storage period groups. FFPE samples were divided into two groups by 340 days, the median storage period. Similarly, fresh-frozen samples were divided by 427 days. [file Image_1.tiff]

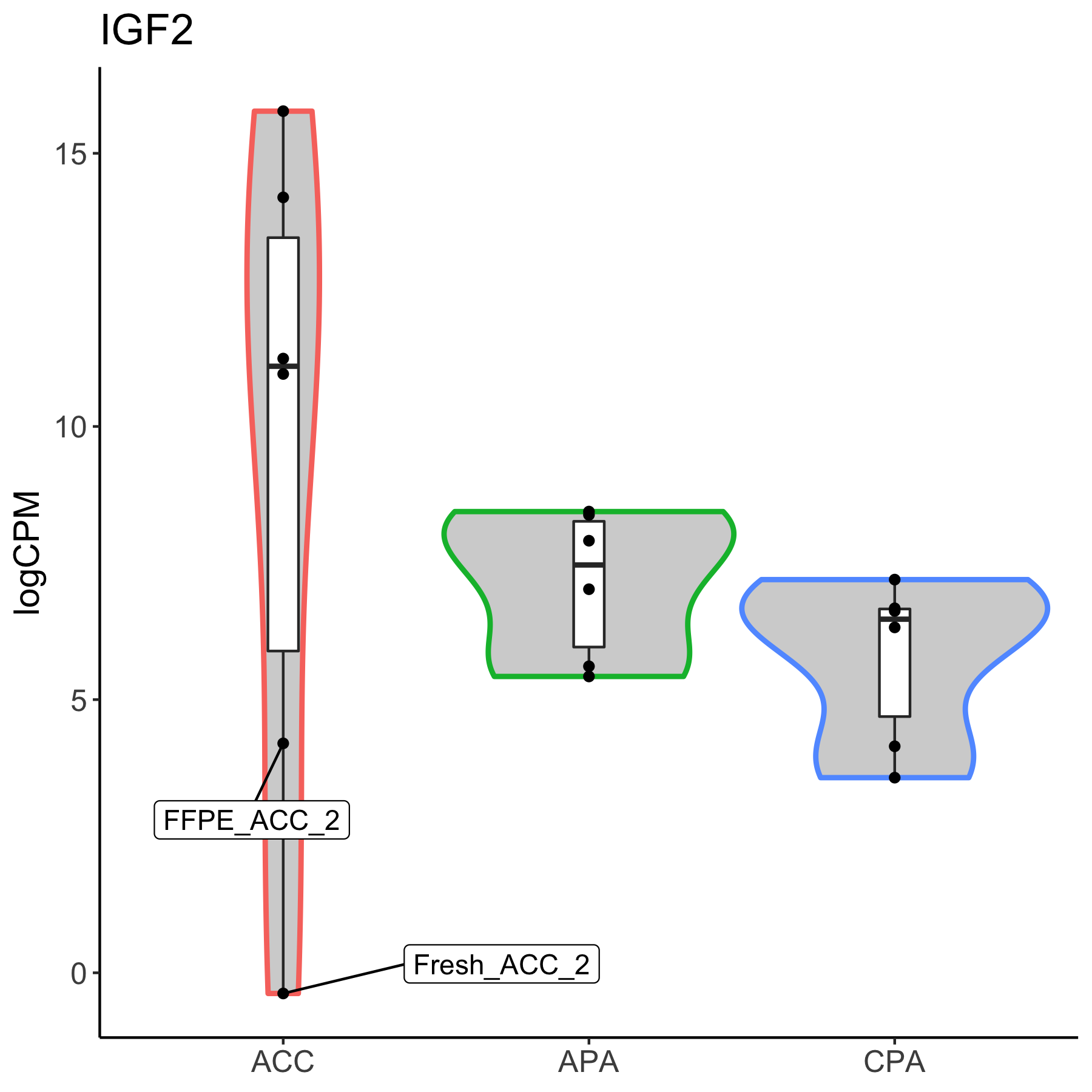

Supplement: Supplementary Figure 2 — Distribution of the logCPM values of IGF2. Dots; each sample. [file Image_2.tiff]

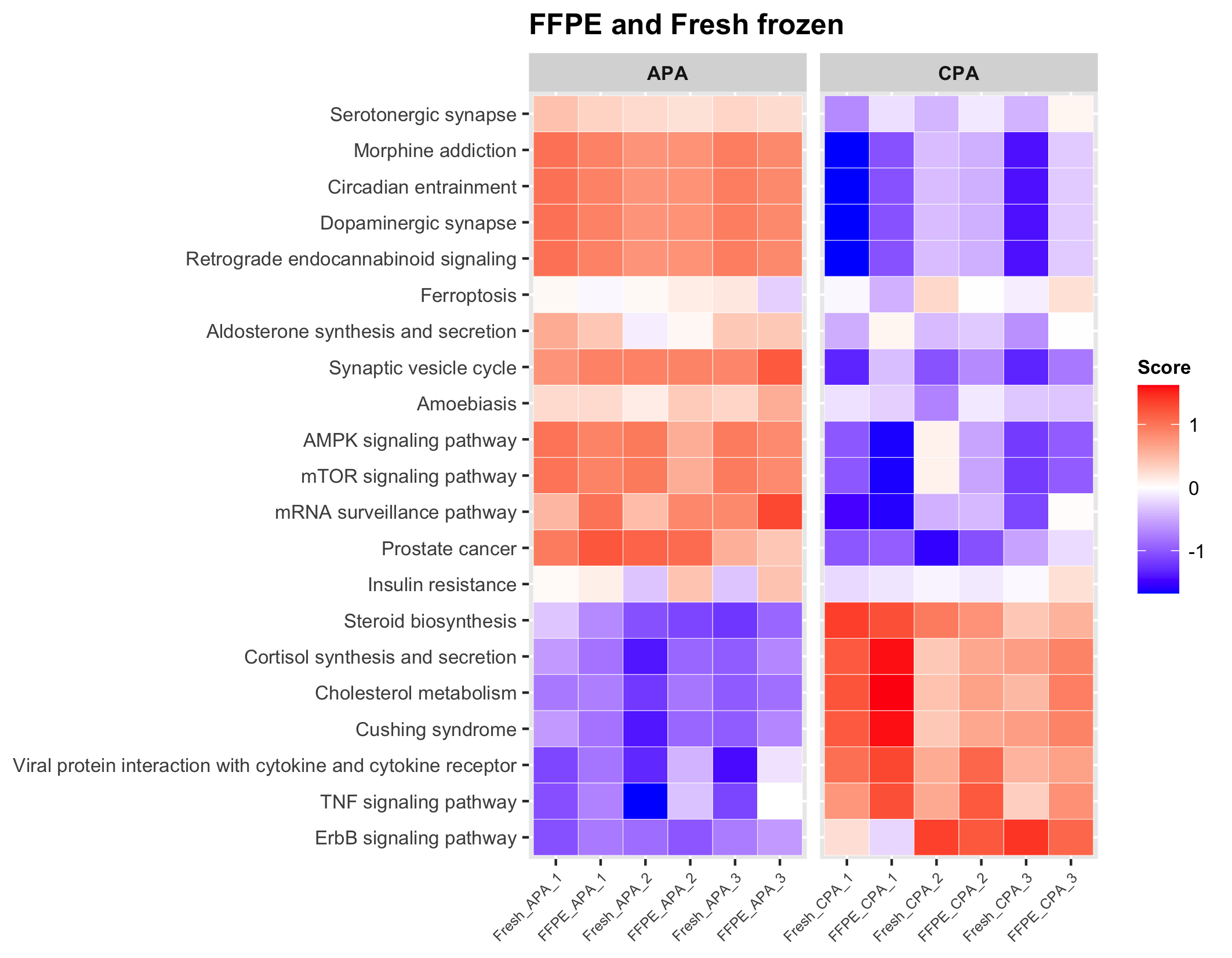

Supplement: Supplementary Figure 3 — Heatmap showing the results of KEGG pathway analysis of DEGs between APA and CPA (common to FFPE and fresh frozen samples). Score; the agglomerated z score of each enriched KEGG pathway per sample. [file Image_3.tiff]
